# A Price Too High: Injury and Assault among Delivery Gig Workers in New York City

**DOI:** 10.1007/s11524-024-00873-9

**Published:** 2024-04-29

**Authors:** Zoey Laskaris, Mustafa Hussein, Jim P. Stimpson, Emilia F. Vignola, Zach Shahn, Nevin Cohen, Sherry Baron

**Affiliations:** 1 Barry Commoner Center for Health and the Environment, Queens College City University of New York, Queens, NY USA; 2 Department of Health Policy and Management, City University of New York Graduate School of Public Health and Health Policy, New York City, NY USA; 3 Peter O’Donnell Jr. School of Public Health, University of Texas Southwestern Medical Center, Dallas, TX USA; 4 Department of Epidemiology, University of Washington School of Public Health, Seattle, WA USA; 5 Department of Epidemiology and Biostatistics, City University of New York Graduate School of Public Health and Health Policy, New York City, NY USA

**Keywords:** Gig economy, Platform work, Food delivery, Occupational health, Income dependence, Injury, Assault, Occupational health disparities

## Abstract

The occupational health burden and mechanisms that link gig work to health are understudied. We described injury and assault prevalence among food delivery gig workers in New York City (NYC) and assessed the effect of job dependence on injury and assault through work-related mechanisms and across transportation modes (electric bike and moped versus car). Data were collected through a 2022 survey commissioned by the NYC Department of Consumer and Worker Protection among delivery gig workers between October and December 2021 in NYC. We used modified Poisson regression models to estimate the adjusted prevalence rate ratio associations between job dependence and injury and assault. Of 1650 respondents, 66.9% reported that food delivery gig work was their main or only job (i.e., fully dependent). About 21.9% and 20.8% of respondents reported being injured and assaulted, respectively. Injury and assault were more than twice as prevalent among two-wheeled drivers, in comparison to car users. Fully dependent respondents had a 1.61 (95% confidence interval (CI) 1.20, 2.16) and a 1.36 (95% CI 1.03, 1.80) times greater prevalence of injury and assault, respectively, than partially dependent respondents after adjusting for age, sex, race and ethnicity, language, employment length, transportation mode, and weekly work hours. These findings suggest that fully dependent food delivery gig workers, especially two-wheeled riders, are highly vulnerable to the negative consequences of working conditions under algorithmic management by the platforms. Improvements to food delivery gig worker health and safety are urgently needed, and company narratives surrounding worker autonomy and flexibility need to be revisited.

**Supplementary Information:**

The online version contains supplementary material available at 10.1007/s11524-024-00873-9.

## Introduction

Gig work is a form of non-standard employment in which digital platforms (“apps”) link workers and customers to perform discrete tasks such as ride-hailing or food delivery [1]. An estimated 16% of all workers in the United States have ever performed some gig work [2]. Such jobs are often precarious, with low pay and limited protections, resulting in stress and risk-taking that adversely affect worker health, safety, and well-being [3, 4]. Moreover, these hazards likely widen health disparities, as gig workers are disproportionately from racialized minority, immigrant, and low-income groups [5, 6], yet the occupational health burden of gig work and the mechanisms that link gig work to health are largely understudied and poorly understood.

The rapid growth of food delivery gig work, especially following the onset of the COVID-19 pandemic [7], makes it a particularly instructive setting to assess how gig work shapes health and health equity. Food delivery platform companies (e.g., DoorDash, Uber Eats, Grubhub) engage hundreds of thousands of US workers as independent contractors delivering meals [3]. In their promotional materials, they advertise this work as a low-barrier and flexible opportunity to supplement income. However, emerging evidence suggests that a large portion of food delivery workers are fully dependent on the platforms as their main job and income source, making them especially vulnerable to injury and harm in ways unique to platform work [3, 8–11].

Platforms use autonomous computer algorithms to maximize productivity and manage employer-worker relationships [3, 12]. Algorithms assign tasks, set work pace, and use surge pricing and other nudges to influence worker behaviors [13, 14]. These levers, often invisible to workers, shape labor conditions, including work intensity, income security, and decision authority [10, 14–16]. Evidence suggests that work-related factors, such as time pressure, high job demands, piece rate pay, and schedule irregularity increase the risk of occupational injuries by contributing to stress, fatigue, anger, and risk-taking behaviors [10, 17–20].

The health consequences of platforms’ algorithmic management likely hinge on workers’ level of dependence on platform work [10, 11, 21–24], which is a proxy of intersecting markers of social position [25]. Minoritized and immigrant groups with fewer formal credentials, household resources, and standard employment prospects are more likely to be fully dependent on platform-based work and thus more likely to experience its negative effects than to accrue its flexibility benefits [3]. Few studies, however, have focused on dependence as a risk factor of occupational injury or as a mechanism contributing to existing occupational health disparities.

In New York City (NYC), platform-based food delivery orders have steadily increased since the 2010s with a surge in orders during the COVID-19 pandemic [8, 26]. The most recent data suggest that NYC is home to approximately 61,000 food delivery gig workers earning an estimated $4.03 per hour without tips prior to a recent policy establishing a $17.96 minimum hourly pay rate [8]. Importantly, most of these workers are fully dependent on food delivery gig work as their main or only source of income, according to a study on the pay and working conditions of food delivery gig workers in NYC commissioned by the NYC Department of Consumer and Worker Protection (DCWP) [8]. The NYC-DCWP also found that the rate of fatal occupational injuries among two-wheeled food delivery gig workers (36 fatalities per 100,000 full-time equivalent (FTE) workers) far exceeded the equivalent rate in the construction industry (7 fatalities per 100,000 FTE), which has, historically, held the highest fatality rate of any industry in NYC [27]. In addition to fatal injuries, the study revealed a high prevalence of non-fatal occupational injuries and assaults, especially among electric bike (e-bike) and moped drivers [8].

We obtained more detailed survey data from the NYC-DCWP to expand the findings in their report with a focus on food delivery gig workers’ injuries and assaults. Our specific objectives were to (1) describe the prevalence of injury and assault among platform-based food delivery workers by level of job dependence and sociodemographic (age, gender, race and ethnicity, and English language ability) and work-related (length of employment, usual weekly hours, and mode of transport) characteristics and (2) assess the effect of job dependence on the prevalence of injury and assault through work-related mechanisms, net of main confounders, and across modes of transport (e-bike and moped vs. car).

## Methods

### Sample

For this study, we utilized data that were collected by the NYC-DCWP in June and July 2022. Detailed methods of sample selection and survey dissemination are described in the NYC-DCWP report [8]. Briefly, an online survey was sent via text message and email to an estimated 122,539 unique, NYC-based, restaurant delivery gig workers who had responded to a delivery for a digital platform company (i.e., Uber Eats, Grubhub, DoorDash, Relay, Chowbus, or HungryPanda) between October and December 2021. The contact information that the workers used to sign up for the platforms was provided to the NYC-DCWP by the companies. Study recruitment, consent, and survey forms were provided in Arabic, Bengali, Chinese, English, French, Korean, Russian, Spanish, and Urdu. To minimize participant burden, survey respondents were randomly assigned to complete one of three survey modules covering different topics (vehicle expenses, non-vehicle expenses, and safety and demographics).

The present study was based on the 2150 unique respondents who completed the safety and demographics module of the NYC-DCWP survey and met the department’s inclusion criteria, including being 18 + years of age, freely consenting to participate in the survey, and having worked for a digital platform company. From the 2150 respondents, we included 1650 (76.7%) in the analytic sample (see Supplemental Fig I) and excluded 263 respondents who were no longer working for a digital platform company, 208 who used a form of transportation other than e-bike or moped (walking, “something else”) to avoid small cell sizes, and 29 with missing or redacted data in at least one of the analytic variables.

### Measures

#### Injury and Assault

We categorized respondents as injured if they reported being “injured seriously enough while working for delivery apps that [they] missed work, lost consciousness, or received medical care” *or* reported being injured due to a physical assault that occurred while working for a delivery app. A separate outcome measure for assault was defined as an affirmative response to “Have you ever been physically assaulted while working for a delivery app?” Not all respondents who reported being physically assaulted were also injured during or due to the assault (breakdown provided in the “Results” section).

#### Dependence Level

The following two questions established a respondent’s level of dependence on delivery gig work: “Is app delivery your only job?” and “Is app delivery your main job?” (asked if the respondent reported that app delivery was not their only job). We considered respondents to be *fully dependent* on digital platform work if it comprised their “only job” or “main” of multiple jobs. *Partially dependent* respondents included those who used digital platform work as a side job, reporting that they worked more than one job and did not consider delivery platform work to be their main job.

#### Covariates

Collected sociodemographic characteristics included age, gender, and race and ethnicity (Hispanic, White non-Hispanic, Black non-Hispanic, Asian, and Other). The “Other” category comprised American Indians, Alaska Natives, Pacific Islanders, and non-Hispanic respondents of multiple races. We used the language in which the participant took the survey as a proxy for English language ability; we considered respondents to have limited English language ability if they opted to take the survey in one of the non-English language choices. The survey did not include items on nativity or immigration status.

Work-related characteristics were collected for length of employment, usual weekly hours, and mode of delivery transportation. Length of employment (less than 1 to 4 + years) was defined as the difference between self-reported start month and year of platform delivery work and the month and year in which the survey was completed (June or July 2022) for all respondents who began work on or after January 1, 2019. An employment length of 4 + years was assigned to all respondents who started working in 2018 or earlier. Usual weekly hours (less than 20 to 40 + h) were collected in response to “How many hours per week do you usually work for delivery apps?” Lastly, we categorized the modes of transport respondents reported “usually” using to make deliveries into two groups, e-bike or mopeds and cars. We combined moped users (*n* = 51) and e-bike users (*n* = 630) into one category to avoid small cell sizes and potential misclassification, as the NYC-DCWP posited that many unregistered mopeds were likely misreported as e-bikes [8].

### Statistical Methods

**Fig. 1:**
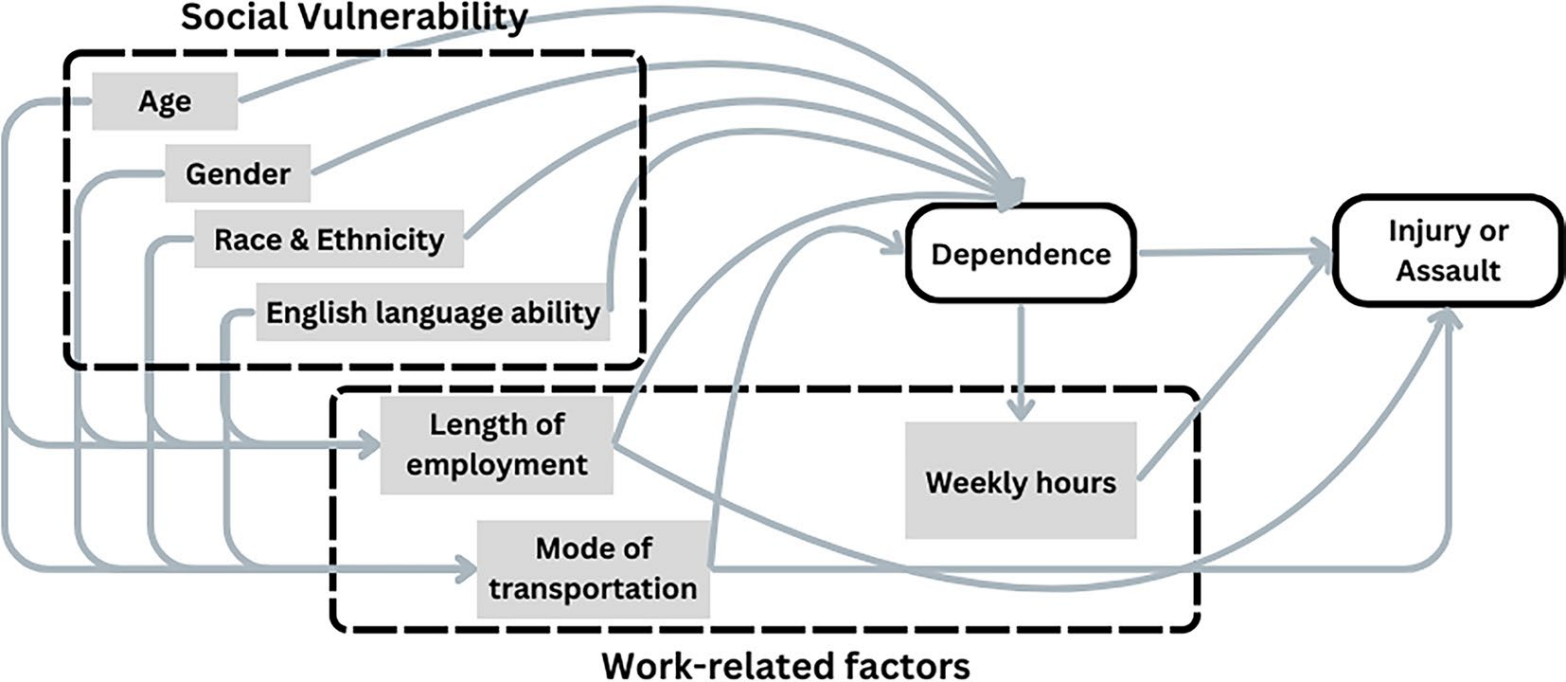
Directed acyclic graph (DAG). The DAG includes sociodemographic and work-related factors measured in the NYC-DCWP survey that are hypothesized to be related to dependence, our main exposure, and injury or assault, our main outcomes, among food delivery gig workers. Regression model adjustments were informed using the DAG

First, we described the prevalence of the outcome measures (injury and assault), the main exposure (dependence), and sociodemographic and work-related covariates for the sample as a whole and stratified by dependence level. We ran modified Poisson regression models with robust standard errors to estimate adjusted prevalence rate ratio associations between dependence and injury and assault. A directed acyclic graph (DAG) informed our model adjustments by helping to identify sociodemographic and work-related characteristics that were hypothesized to be related to dependence and injury and assault (Fig. 1). For each outcome measure, we fit three separate adjusted models; the base model (Model 1) was adjusted for sociodemographic characteristics and length of employment, which are known confounders of the association between dependence and injury or assault. Model 1 offers base estimates of the “total” magnitude of these associations through all mechanisms. Models 2 and 3 stepwise adjusted for mode of transport and usual weekly work hours. While mode of transport is a potential risk factor for injury and assault and, if pre-existent, a driver of dependence and thus a confounder, it may also be a consequence of dependence if, say, fully dependent workers invest in a particular model of transport (e.g., e-bike) to pursue their work. Work hours are plausibly immediate manifestations of dependence and hence mediators of the dependence-outcomes associations. Models 2 and 3 thus offer estimates of the direct effects of dependence on injury and assault through unobserved mechanisms beyond mode and hours, such as earnings, work intensity, and risk-taking behavior. Further, since two- and four-wheeled modes of transport pose very distinct injury threats, we examined the effects of dependence on injury and assault in a version of Model 3 stratified by mode of transport. In a sensitivity analysis, propensity score matching (PSM) was used to estimate the effect of dependence on the outcome measures while explicitly accounting for observed confounding. Graphical methods were used to examine the balance between the treated and untreated groups, and effect estimates from linear versions of Models 1 and 2 adjusted for the same sets of covariates were compared with the PSM results. Given that usual weekly hours may be a consequence of dependence level, it was not included in the PSM models.

## Results

Sample characteristics are described in Table 1. The sample comprised 1650 current (at the time of the survey) platform-based food delivery workers based in NYC. Of these, 66.9% reported that platform-based delivery work was their main or only job (i.e., fully dependent), whereas the rest (33.1%) were partially dependent. While working for a platform company, 21.9% and 20.8% reported being injured and assaulted, respectively. Among the 343 respondents who reported having been assaulted, 39.9% were injured due to the assault (data not shown). Most respondents (68.8%) were between 25 and 44 years of age and male (77.5%). Respondents were racially and ethnically diverse; 50.3% identified as Hispanic, 23.9% as Black (non-Hispanic), and 15.6% as Asian. Approximately one in four respondents (26.7%) had limited English language ability. Although 43.9% of the respondents started working as a food delivery gig worker between 2020 and 2022, almost one-quarter of respondents (22.8%) had worked for 4 + years. There was also a wide distribution in usual weekly hours; 37.4% and 28.2% reported working less than 20 h and 40 or more hours per week, respectively. The sample was closely divided among car (58.7%) and e-bike or moped users (41.3%).

**Table 1:** Descriptive characteristics of the analytic sample (*n* = 1650) by level of dependence on app-based delivery work, NYC-DCWP survey (2022)

	Total	Dependence	
		Main job	Side job
	*n* (%)	*n* (%)	*n* (%)
Dependence			
Main job	1104 (66.9)	1104 (100.0)	–
Side job	546 (33.1)	–	546 (100.0)
Ever injured (from accident or assault)			
No	1289 (78.1)	799 (72.4)	490 (89.7)
Yes	361 (21.9)	305 (27.6)	56 (10.3)
Ever assaulted while working			
No	1307 (79.2)	825 (74.7)	482 (88.3)
Yes	343 (20.8)	279 (25.3)	64 (11.7)
Age (years)			
18–24	248 (15.0)	178 (16.1)	70 (12.8)
25–34	665 (40.3)	446 (40.4)	219 (40.1)
35–44	470 (28.5)	310 (28.1)	160 (29.3)
45 and older	267 (16.2)	170 (15.4)	97 (17.8)
Gender			
Male	1279 (77.5)	885 (80.2)	394 (72.2)
Female	371 (22.5)	219 (19.8)	152 (27.8)
Race and ethnicity			
Hispanic	830 (50.3)	557 (50.5)	273 (50.0)
White (non-Hispanic)	127 (7.7)	81 (7.3)	46 (8.4)
Black (non-Hispanic)	394 (23.9)	240 (21.7)	154 (28.2)
Asian	257 (15.6)	197 (17.8)	60 (11.0)
Other	42 (2.5)	29 (2.6)	13 (2.4)
English language ability			
Proficient	1209 (73.3)	767 (69.5)	442 (81.0)
Limited	441 (26.7)	337 (30.5)	104 (19.0)
Length of employment			
Less than 1 year	314 (19.0)	206 (18.7)	108 (19.8)
1 to 2 years	411 (24.9)	253 (22.9)	158 (28.9)
2 to 3 years	381 (23.1)	263 (23.8)	118 (21.6)
3 to 4 years	168 (10.2)	114 (10.3)	54 (9.9)
4 + years	376 (22.8)	268 (24.3)	108 (19.8)
Mode of transport			
Car	969 (58.7)	566 (51.3)	403 (73.8)
Moped, e-bike	681 (41.3)	538 (48.7)	143 (26.2)
Usual weekly hours			
Less than 20 h	617 (37.4)	264 (23.9)	353 (64.7)
20–39 h	568 (34.4)	401 (36.3)	167 (30.6)
40 or more hours	465 (28.2)	439 (39.8)	26 (4.8)
Total	1650 (100.0)	1104 (100)	546 (100.0)

Except for age, sample characteristics differed by dependence level (Table 1). Injury and assault were reported among 27.6% and 25.3% of the fully dependent respondents, respectively, in comparison to 10.3% and 11.7% of the partially dependent respondents, respectively. Fully dependent respondents were relatively more likely to be male, Asian, and to have limited English proficiency, than partially dependent respondents. Partially dependent respondents comprised larger proportions of women, Black (non-Hispanic), and English-speaking respondents. Moped and e-bike use was more common among fully dependent (48%) in comparison to partially dependent (26.2%) respondents.

Tables 2, 3, and 4 provide results from adjusted modified Poisson regression models (bivariable results are provided in Supplemental Table I). A positive association between dependence and injury was observed in Models 1, 2, and 3 (Table 2). In Model 1, adjusted for the main sociodemographic confounders and length of employment, fully dependent respondents had a 2.39 (95% confidence interval (95% CI) 1.83–3.12) times greater prevalence of injury in comparison to partially dependent respondents. This association attenuated by 16%, down to an adjusted prevalence ratio (aPR) of 2.02 (95% CI 1.54–2.64) with further adjustment for the mode of transport (Model 2). In the fully adjusted model (Model 3), which further controlled for work hours, the prevalence of injury among fully dependent respondents was 1.61 (95% CI 1.20–2.16) times that of partially dependent respondents. We observed a similar pattern for assault (Table 3), where fully dependent respondents had a 75% (95% CI 1.36, 2.25) higher prevalence of assault in Model 1, attenuating to 36% (95% CI 1.03, 1.80) in Model 3 after adjusting for mode of transport and hours, relative to their partially dependent counterparts. Our PSM results on the additive scale are also consistent with these findings (Suppl. Table II).

**Table 2:** Adjusted prevalence ratios for *injury* by dependence on app-based delivery work and sociodemographic and work-related covariates, NYC-DCWP survey (2022), *n* = 1650

	Injury^a^		
	Model 1^b^	Model 2^b^	Model 3^b^
	aPR [95% CI]	aPR [95% CI]	aPR [95% CI]
Dependence			
Side job	1 [1.00, 1.00]	1 [1.00, 1.00]	1 [1.00, 1.00]
Main job	2.39*** [1.83, 3.12]	2.02*** [1.54, 2.64]	1.61** [1.20, 2.16]
Age (years)			
18–24	1 [1.00, 1.00]	1 [1.00, 1.00]	1 [1.00, 1.00]
25–34	0.85 [0.67, 1.09]	0.91 [0.72, 1.16]	0.87 [0.69, 1.11]
35–44	0.76 [0.58, 1.01]	0.85 [0.65, 1.12]	0.82 [0.63, 1.07]
45 and older	0.75 [0.54, 1.04]	0.97 [0.71, 1.33]	0.93 [0.68, 1.28]
Gender			
Male	1 [1.00, 1.00]	1 [1.00, 1.00]	1 [1.00, 1.00]
Female	0.61*** [0.46, 0.81]	0.76 [0.57, 1.01]	0.84 [0.63, 1.13]
Race and ethnicity			
Hispanic	1.07 [0.75, 1.53]	0.92 [0.64, 1.32]	0.91 [0.64, 1.30]
White (non-Hispanic)	1 [1.00, 1.00]	1 [1.00, 1.00]	1 [1.00, 1.00]
Black (non-Hispanic)	0.97 [0.66, 1.44]	0.85 [0.58, 1.26]	0.84 [0.57, 1.23]
Asian	1.22 [0.83, 1.80]	1.06 [0.71, 1.56]	1 [0.68, 1.47]
Other	1.56 [0.91, 2.68]	1.45 [0.86, 2.44]	1.6 [0.95, 2.71]
English language ability			
Proficient	1 [1.00, 1.00]	1 [1.00, 1.00]	1 [1.00, 1.00]
Limited	1.2 [0.98, 1.47]	1.08 [0.89, 1.31]	1.05 [0.87, 1.28]
Length of employment			
Less than 1 year	1 [1.00, 1.00]	1 [1.00, 1.00]	1 [1.00, 1.00]
1 to 2 years	0.83 [0.60, 1.16]	0.86 [0.63, 1.19]	0.87 [0.64, 1.20]
2 to 3 years	1.41* [1.06, 1.87]	1.42* [1.07, 1.88]	1.36* [1.03, 1.80]
3 to 4 years	1.55** [1.12, 2.16]	1.65** [1.20, 2.28]	1.57** [1.15, 2.16]
4 + years	1.46* [1.09, 1.95]	1.46** [1.10, 1.94]	1.40* [1.06, 1.86]
Mode of transport			
Car		1 [1.00, 1.00]	1 [1.00, 1.00]
Moped, e-bike		2.34*** [1.90, 2.88]	2.32*** [1.89, 2.85]
Usual weekly hours			
Less than 20 h			1 [1.00, 1.00]
20–39 h			1.23 [0.94, 1.62]
40 or more hours			1.81*** [1.39, 2.36]

*aPR* adjusted prevalence ratio

*P* value = * < 0.05, ** < 0.01, *** < 0.001

^a^Injury is defined as affirmative responses to “Have you been injured seriously enough while working for delivery apps that you missed work, lost consciousness, or received medical care” or “Were you injured in any of the assaults?,” among those who reported being physically assaulted while working for a delivery app

^b^Results were derived from modified Poisson regression models with robust standard errors

**Table 3:** Adjusted prevalence ratios for *assault* by dependence on app-based delivery work and sociodemographic and work-related covariates, NYC-DCWP survey (2022), *n* = 1650

	Assault^a^		
	Model 1^b^	Model 2^b^	Model 3^b^
	aPR [95% CI]	aPR [95% CI]	aPR [95% CI]
Dependence			
Side job	1.0 [1.00, 1.00]	1 [1.00, 1.00]	1.0 [1.00, 1.00]
Main Job	1.75*** [1.36, 2.25]	1.50** [1.16, 1.93]	1.36* [1.03, 1.80]
Age (years)			
18–24	1 [1.00, 1.00]	1 [1.00, 1.00]	1 [1.00, 1.00]
25–34	0.9 [0.69, 1.17]	0.95 [0.74, 1.23]	0.94 [0.72, 1.21]
35–44	0.81 [0.61, 1.07]	0.9 [0.68, 1.18]	0.88 [0.67, 1.16]
45 and older	0.64* [0.45, 0.91]	0.82 [0.58, 1.16]	0.81 [0.57, 1.14]
Gender			
Male	1 [1.00, 1.00]	1 [1.00, 1.00]	1 [1.00, 1.00]
Female	0.47*** [0.34, 0.66]	0.58** [0.41, 0.81]	0.60** [0.43, 0.85]
Race and ethnicity			
Hispanic	1.06 [0.72, 1.55]	0.91 [0.62, 1.34]	0.91 [0.62, 1.33]
White (non-Hispanic)	1 [1.00, 1.00]	1 [1.00, 1.00]	1 [1.00, 1.00]
Black (non-Hispanic)	0.79 [0.51, 1.23]	0.69 [0.45, 1.07]	0.69 [0.44, 1.06]
Asian	1.09 [0.72, 1.65]	0.95 [0.63, 1.44]	0.93 [0.61, 1.40]
Other	1.33 [0.73, 2.43]	1.19 [0.65, 2.18]	1.24 [0.68, 2.28]
English language ability			
Proficient	1 [1.00, 1.00]	1 [1.00, 1.00]	1 [1.00, 1.00]
Limited	1.87*** [1.54, 2.28]	1.70*** [1.40, 2.06]	1.68*** [1.38, 2.04]
Length of employment			
Less than 1 year	1 [1.00, 1.00]	1 [1.00, 1.00]	1 [1.00, 1.00]
1 to 2 years	1.04 [0.74, 1.46]	1.06 [0.76, 1.47]	1.06 [0.76, 1.48]
2 to 3 years	1.33 [0.97, 1.83]	1.33 [0.97, 1.81]	1.31 [0.96, 1.78]
3 to 4 years	1.84*** [1.30, 2.62]	1.94*** [1.37, 2.74]	1.90*** [1.34, 2.68]
4 + years	1.80*** [1.33, 2.45]	1.80*** [1.33, 2.43]	1.79*** [1.32, 2.41]
Mode of transport			
Car		1 [1.00, 1.00]	1 [1.00, 1.00]
Moped, e-bike		2.12*** [1.71, 2.62]	2.11*** [1.70, 2.62]
Usual weekly hours			
Less than 20 h			1 [1.00, 1.00]
20–39 h			1.04 [0.81, 1.34]
40 or more hours			1.28* [1.00, 1.64]

*aPR* adjusted prevalence ratio

*P* value = * < 0.05, ** < 0.01, *** < 0.001

^a^Assault is defined as an affirmative response to “Have you ever been physically assaulted while working for a delivery app?”

^b^Results were derived from modified Poisson regression models with robust standard errors

**Table 4:** Adjusted prevalence ratios for injury and assault by mode of transport, NYC-DCWP survey, *n* = 1650

	Injury^a^		Assault^b^	
	Moped and e-bike	Car	Moped and e-bike	Car
	aPR [95% CI]^c^	aPR [95% CI]^c^	aPR [95% CI]^c^	aPR [95% CI]^c^
Dependence				
Side job	1 [1.00, 1.00]	1 [1.00, 1.00]	1 [1.00, 1.00]	1 [1.00, 1.00]
Main job	1.29 [0.91, 1.83]	2.10** [1.30, 3.41]	1.39 [0.96, 2.01]	1.35 [0.90, 2.05]
Age (years)				
18–24	1 [1.00, 1.00]	1 [1.00, 1.00]	1 [1.00, 1.00]	1 [1.00, 1.00]
25–34	0.84 [0.64, 1.10]	0.95 [0.58, 1.55]	0.94 [0.70, 1.25]	0.93 [0.53, 1.63]
35–44	0.85 [0.63, 1.14]	0.78 [0.45, 1.35]	0.95 [0.70, 1.29]	0.75 [0.41, 1.37]
45 and older	1.1 [0.77, 1.57]	0.82 [0.45, 1.47]	0.88 [0.57, 1.36]	0.67 [0.36, 1.27]
Gender				
Male	1 [1.00, 1.00]	1 [1.00, 1.00]	1 [1.00, 1.00]	1 [1.00, 1.00]
Female	0.75 [0.48, 1.17]	0.96 [0.64, 1.44]	0.72 [0.44, 1.16]	0.56* [0.35, 0.92]
Race and ethnicity				
Hispanic	1.13 [0.68, 1.89]	0.8 [0.48, 1.33]	0.97 [0.55, 1.71]	0.86 [0.51, 1.45]
White (non-Hispanic)	1 [1.00, 1.00]	1 [1.00, 1.00]	1 [1.00, 1.00]	1 [1.00, 1.00]
Black (non-Hispanic)	1.18 [0.69, 2.01]	0.58 [0.33, 1.03]	0.78 [0.42, 1.46]	0.58 [0.31, 1.08]
Asian	1.2 [0.70, 2.06]	0.98 [0.55, 1.74]	0.98 [0.54, 1.78]	0.9 [0.49, 1.66]
Other	2.18* [1.10, 4.35]	0.99 [0.42, 2.34]	1.31 [0.58, 2.96]	1.05 [0.41, 2.69]
English language ability				
Proficient	1 [1.00, 1.00]	1 [1.00, 1.00]	1 [1.00, 1.00]	1 [1.00, 1.00]
Limited	1.21 [0.97, 1.50]	0.73 [0.46, 1.18]	1.67*** [1.34, 2.08]	1.74** [1.16, 2.60]
Length of employment				
Less than 1 year	1 [1.00, 1.00]	1 [1.00, 1.00]	1 [1.00, 1.00]	1 [1.00, 1.00]
1 to 2 years	1.01 [0.70, 1.46]	0.59 [0.31, 1.12]	1.07 [0.74, 1.54]	1.11 [0.52, 2.33]
2 to 3 years	1.31 [0.95, 1.81]	1.45 [0.88, 2.41]	1.16 [0.82, 1.63]	1.77 [0.89, 3.52]
3 to 4 years	1.58* [1.09, 2.29]	1.66 [0.95, 2.90]	1.38 [0.92, 2.09]	3.40*** [1.70, 6.81]
4 + years	1.41* [1.01, 1.96]	1.44 [0.85, 2.45]	1.44* [1.03, 2.01]	3.01** [1.53, 5.92]
Usual weekly hours				
Less than 20 h	1 [1.00, 1.00]	1 [1.00, 1.00]	1 [1.00, 1.00]	1 [1.00, 1.00]
20–39 h	1.12 [0.81, 1.53]	1.48 [0.92, 2.39]	0.95 [0.70, 1.30]	1.29 [0.85, 1.95]
40 or more hours	1.70*** [1.26, 2.28]	2.02** [1.22, 3.34]	1.28 [0.96, 1.71]	1.3 [0.82, 2.06]

*aPR* adjusted prevalence ratio

*P* value = * < 0.05, ** < 0.01, *** < 0.001

^a^Injury is defined as affirmative responses to “Have you been injured seriously enough while working for delivery apps that you missed work, lost consciousness, or received medical care” or “Were you injured in any of the assaults?,” among those who reported being physically assaulted while working for a delivery app

^b^Assault is defined as an affirmative response to “Have you ever been physically assaulted while working for a delivery app?”

^c^Results were derived from modified Poisson regression models with robust standard errors

Both injury and assault were more than twice as prevalent among e-bike or moped drivers in comparison to car users in the fully adjusted models (aPR 2.32 for injury; 95% CI 1.89, 2.85; Table 2; 2.12 for assault, 95% CI 1.71, 2.62; Table 3). Model results stratified by mode of transport are presented in Table 4. Among e-bike or moped users, who experience already a high prevalence of injury, having delivery gig work as one’s main job was associated with only marginal increases in the likelihood of injury (aPR 1.29; 95% CI 0.91, 1.83; Table 4). Among car users only, being fully dependent on delivery gig work was associated with a 2.10 times greater prevalence of injury (95% CI 1.30, 3.41), net of sociodemographic confounders, length of employment, and work hours.

Having limited English language ability was significantly associated with an increased prevalence of assault (aPR 1.68; 95% CI 1.38, 2.04; Table 3), but not injury (aPR 1.05; 95% CI 0.87, 1.28; Table 2) in the fully adjusted models overall and across mode of transport groups (Table 4). Lastly, females appeared to be significantly less likely to experience assault than males in fully adjusted models (e.g., in Table 4, aPR 0.56; 95% CI 0.35, 0.92).

## Discussion

We examined predictors of occupational injury and assault among a population of mostly Hispanic, Black, and Asian food delivery gig workers in NYC using unique data from the NYC-DCWP survey. Two key findings emerged from our analysis. First, among the 1650 respondents in our sample, most (67%) were fully dependent on platform-based work and over 21% reported being injured or assaulted while on the job. Second, full dependence on platform work was significantly associated with a greater probability of injury and assault, by 61% and 36%, respectively, relative to partial dependence, even after adjusting for work experience, delivery mode, weekly hours, and sociodemographics. Together, these findings point to the clear dangers associated with platform-based delivery work, especially for two-wheeled drivers, and reinforce the importance of dependence as a key moderator of risk. They also challenge company narratives that most people engage in gig delivery work as a supplemental, “flexible” form of paid work.

The diversity in levels of economic dependence found in our study population is a distinct characteristic of platform work associated with platform companies’ willingness to accept workers irrespective of their other work commitments [11]. The high proportion of fully dependent workers in our and in other study samples [10, 26], along with the fact that 40% of fully dependent respondents worked 40 + hours per week, raises questions regarding whether workers are able to benefit from the flexibility that food delivery companies advertise.

Importantly, dependence on platform-based work was an indicator of high social vulnerability in our sample. Although most respondents were of a racialized minority population, fully dependent respondents were less likely to use cars and more likely to have limited English language proficiency than partially dependent respondents. In this context, the limited (or non-existent) access to worker protections, such as workers’ compensation insurance, among high-risk dependent respondents can exacerbate existing health disparities; foreign-born workers of low socio-economic position may be less able to cope with the acute and downstream consequences of a work-related injury (e.g., lost wages, medical expenses, difficulty paying important bills, and threats to housing).

The positive association between dependence and work injury among food delivery gig workers echoes recent research on this emerging topic. Using qualitative data, Schor et al. explored the relationship between dependence level and work risks and concluded that those with greater economic insecurity and who were more dependent on the income from the platform job were less likely to feel in control of key aspects of their work, including flexibility to choose which times, places, specific tasks, and platforms maximize compensation [28]. Similar to our findings, Jing et al. found that dependence on platform-based work was associated with 23% greater odds of work injury compared to non-dependence in a study among platform-based food delivery workers in China [10]. They also found that workload was a mediator on the pathway between dependence and injury [10]. Further research is needed to replicate these findings and identify other mechanisms for how higher levels of dependence place workers at greater risk for injuries and assaults.

Research on the health effects of algorithmic management suggests other avenues in which dependence on platform-based work may precipitate occupational injury and assault. Specific features of algorithmic management, used by platform-based food delivery companies for performance monitoring, scheduling, compensation, and hiring and termination, have the potential to contribute to income insecurity, schedule instability, isolation, and limited decision authority, which are linked with feelings of stress, fatigue, anxiety, and anger [3]. In this state of heightened stress, combined with a high workload, workers may be more easily distracted and likely to engage in risk-taking behaviors, such as speeding or not waiting at traffic lights, which are behaviors reported among food delivery workers [9, 10] and risk factors of traffic collisions and accidents [17]. Moreover, platform companies incentivize workers with bonuses to make deliveries during inclement weather when delivery demand is high and road conditions are unsafe [10]. Other risk factors of injury among two-wheeled delivery drivers include insufficient infrastructure for safe cycling, the use of damaged equipment, and the lack of adequate personal protective equipment (PPE) (e.g., helmets, lights, protective clothing). In all of these scenarios, platform companies are not legally obligated to help workers recoup expenses related to medical treatment, lost wages, or stolen and damaged equipment given their ability to (mis)classify workers as independent contractors rather than employees [1, 29].

The mechanisms leading to assault among food delivery workers may be similar to those leading to injury but are not as well studied. There is evidence that bike theft is a common catalyst of assault in addition to reports of mistreatment and harassment from employees and customers at pickup and drop-off delivery locations [10, 26]. Our finding that limited English language ability was positively associated with assault, but not injury, suggests that discrimination among foreign-born workers should be considered a potential risk factor of assault in future studies.

### Strengths and Limitations

This study used existing data from the NYC-DCWP survey. The collection of data from a population of workers who are otherwise invisible to most occupational safety and health data surveillance systems is a strength of the parent study on which our analyses are based. Also, while our sample comes from one segment of the gig economy in NYC, our findings likely apply to cities with similar dense urban cores and more broadly to workers under similar “gig” working conditions.

Despite the strengths of the NYC-DCWP survey, its design posed several important limitations to our study. Our analysis was based on a convenience sample and may not be representative of the underlying population of food delivery gig workers in NYC, which has yet to be fully characterized. Given the cross-sectional design, former workers were not asked questions related to job dependence and, therefore, were excluded from our sample, which could lead to an under- or overestimate of the association between dependence and injury and assault. Since the NYC-DCWP survey only included platform workers, we are unable to distinguish occupational risks that are inherent in food delivery work from those that are associated with the platformization of the food delivery industry. We used the items related to “main job” as a marker of dependence level, though dependence is a more complex construct that deserves further research. The term “main job” was not defined in the survey question and may have been interpreted in different ways. Relatedly, the survey did not include data on other key drivers of dependence (e.g., household composition, earnings and resources, spousal employment, and education). Future research will need to further develop the theory and drivers of dependence and its relationship to health. Finally, our results may not be generalizable to food delivery gig workers in urban settings with less e-bike and moped density than in NYC; however, we did find that, among car riders only, dependence remained a significant risk factor of injury.

### Recommendations and Future Research

Our findings provide evidence in support of labor protection laws and policies designed to address work conditions, urban transportation issues (e.g., physical infrastructure and traffic enforcement), and platform company-specific practices related to the (mis)classification of independent contractors and the design of algorithms. Immediate efforts to improve worker safety can draw from the core elements of safety management programs (e.g., management commitment, worker participation, hazard identification and assessment, hazard prevention and control, education and training, program evaluation) described by the US Department of Labor Occupational Safety and Health Administration [30] and food delivery safety guidelines by local authorities in the US [31] and in Australia [32]. At a minimum, platform-based companies should be required to ensure that workers have adequate PPE and report work-related injuries, illnesses, and fatalities. Improved surveillance systems can be used to both monitor the rates of injury and assault among platform-based workers and assess the efficacy of policies, such as the recent NYC law governing minimum pay, delivery distances, and other aspects of workers’ safety [33]. Future research should include longitudinal and alternative study designs to better understand the work-related mechanisms on the pathway between dependence and occupational injury, identify circumstances leading to assault, and describe downstream physical, mental, and economic consequences associated with occupational hazards among platform-based workers.

## Conclusion

In NYC and other cities, food delivery gig work is more likely to be a worker’s main source of income than a “flexible” side hustle, despite platform company narratives. The results of this and existing studies suggest that the deliver-at-all-cost reality that is needed to survive financially as a fully dependent food delivery gig worker creates a perfect storm for occupational injury and, to a lesser extent, assault. Further research is needed to understand the full extent and causes of occupational injury and assault among on-demand gig workers and the spectrum of modifiable work-related mechanisms contributing to adverse health outcomes. Finally, further development and implementation of policies designed to hold platform-based companies accountable for the health and safety of their workers are urgently needed. Without them, platform-based companies will remain immune to the consequences of work-related hazards associated with gig work, perpetuating long-standing health disparities among a largely immigrant and socioeconomically disadvantaged worker population.

### Supplementary Information

Below is the link to the electronic supplementary material.

Supplementary file1 (DOCX 164 KB)

## Data Availability

The survey data used in this study is available from the New York City Department of Consumer and Worker Protection at the following web address: https://www.nyc.gov/site/dca/workers/Delivery-Worker-Public-Hearing-Minimum-Pay-Rate.page.

## Declarations

Sherry Baron was a paid consultant to the NYC DCWP in developing the survey questions and in generating the initial analysis of the injury data that was included in the NYC DCWP report (https://www.nyc.gov/assets/dca/downloads/pdf/workers/Delivery-Worker-Study-November-2022.pdf).
