# Supplementary material for: A Price Too High: Injury and Assault among Delivery Gig Workers in New York City
Source: J Urban Health. 2024 Apr 29;101(3):439–50. doi: 10.1007/s11524-024-00873-9 (PMC11189866; doi:10.1007/s11524-024-00873-9)
Supplement: Supplementary file 1 — Supplementary file1 (DOCX 164 KB) [file 11524_2024_873_MOESM1_ESM.docx]

**SUPPLEMENTARY MATERIALS**

**Title:** A Price Too High: Injury and Assault Among Delivery Gig Workers in New York City

**Authors:**

Zoey Laskaris^1^, Mustafa Hussein^2^, Jim P Stimpson^3^, Emilia F Vignola^4^, Zach Shahn^5^, Nevin Cohen^2^, Sherry Baron^1^

^1^Barry Commoner Center for Health and the Environment, Queens College City University of New York, Queens, NY, USA
^2^Department of Health Policy and Management, City University of New York Graduate School of Public Health and Health Policy, NYC, NY, USA
^3^Peter O'Donnell Jr. School of Public Health, University of Texas Southwestern Medical Center, Dallas, TX, USA
^4^ Department of Epidemiology, University of Washington School of Public Health, Seattle, WA, USA

^5^ Department of Epidemiology and Biostatistics, City University of New York Graduate School of Public Health and Health Policy, NYC, NY, USA

**Supplemental Figure I: Sample flow diagram**

**
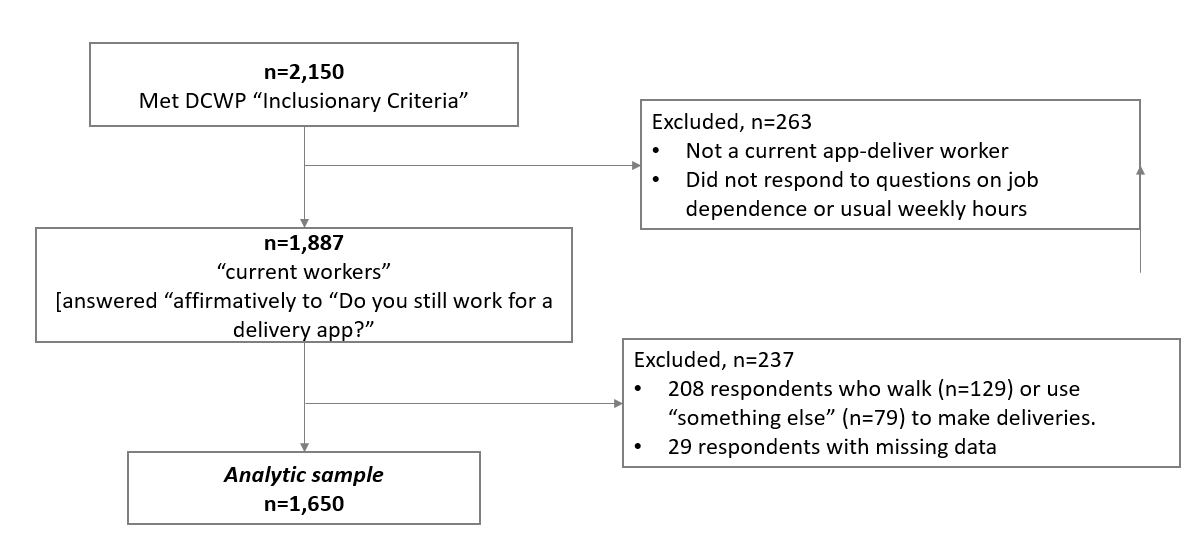
**

**Supplemental Figure II: Balance plots for PSM models**

**
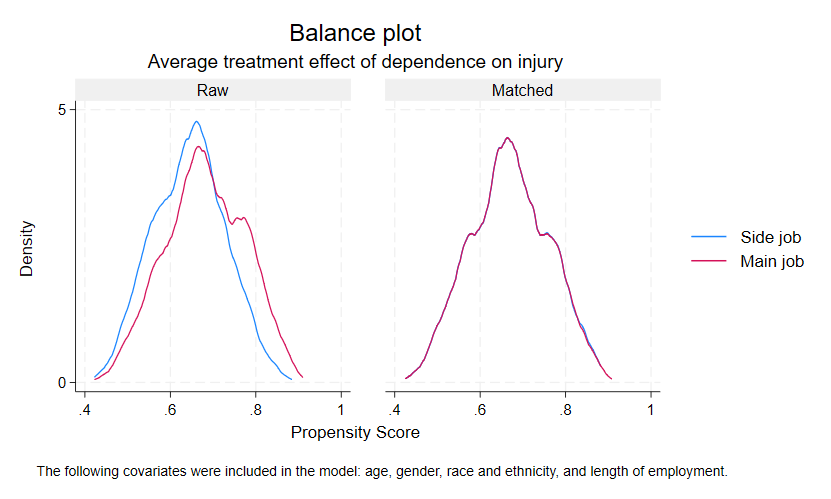

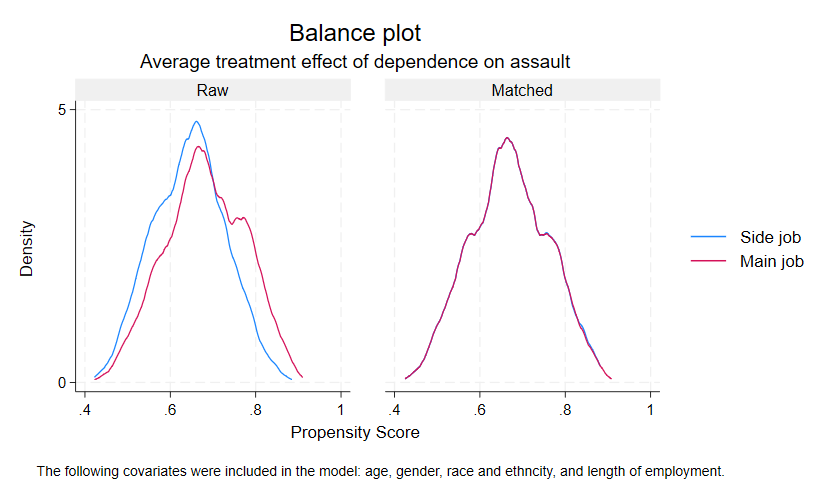
**

**Supplemental Table I: Bivariable prevalence rate ratios for injury and assault from the NYC-DCWP survey (2022)**

|  |  | **INJURY** | **ASSAULT** |
| --- | --- | --- | --- |
|  |  | PR [95% CI] | PR [95% CI] |
| Dependence | |  |  |
|  | Side job | 1 [1.00,1.00] | 1 [1.00,1.00] |
|  | Main job | 2.69*** [2.06,3.51] | 2.16*** [1.68,2.77] |
| Age (years) | |  |  |
|  | 18-24 | 1 [1.00,1.00] | 1 [1.00,1.00] |
|  | 25-34 | 0.86 [0.66,1.11] | 0.96 [0.73,1.26] |
|  | 35-44 | 0.79 [0.60,1.04] | 0.92 [0.69,1.23] |
|  | 45 and older | 0.78 [0.57,1.08] | 0.75 [0.52,1.06] |
| Gender |  |  |  |
|  | Male | 1 [1.00,1.00] | 1 [1.00,1.00] |
|  | Female | 0.50*** [0.38,0.67] | 0.37*** [0.26,0.52] |
| Race & Ethnicity | |  |  |
|  | Hispanic | 1.13 [0.77,1.64] | 1.3 [0.88,1.92] |
|  | White (non-Hispanic) | 1 [1.00,1.00] | 1 [1.00,1.00] |
|  | Black (non-Hispanic) | 0.89 [0.59,1.34] | 0.7 [0.45,1.10] |
|  | Asian | 1.38 [0.92,2.07] | 1.4 [0.91,2.14] |
|  | Other | 1.57 [0.89,2.79] | 1.31 [0.68,2.53] |
| English language ability | |  |  |
|  | Proficient | 1 [1.00,1.00] | 1 [1.00,1.00] |
|  | Limited | 1.37** [1.13,1.65] | 2.16*** [1.80,2.59] |
| Length of employment | |  |  |
|  | Less than 1 year | 1 [1.00,1.00] | 1 [1.00,1.00] |
|  | 1 to 2 years | 0.82 [0.59,1.15] | 1.02 [0.72,1.45] |
|  | 2 to 3 years | 1.47* [1.09,1.97] | 1.42* [1.02,1.97] |
|  | 3 to 4 years | 1.60** [1.14,2.25] | 1.87*** [1.31,2.67] |
|  | 4+ years | 1.55** [1.16,2.07] | 1.87*** [1.37,2.54] |
| Usual weekly hours | |  |  |
|  | Less than 20 hrs | 1 [1.00,1.00] | 1 [1.00,1.00] |
|  | 20 - 39 hrs | 1.51** [1.16,1.96] | 1.23 [0.95,1.58] |
|  | 40 or more hrs | 2.82*** [2.22,3.57] | 2.07*** [1.64,2.61] |
| Mode of transport | |  |  |
|  | Car | 1 [1.00,1.00] | 1 [1.00,1.00] |
|  | Moped, E-Bike | 2.82*** [2.32,3.43] | 2.78*** [2.28,3.40] |

P value * <0.05 **<0.01 **<0.001

Footnote: Results were derived from unadjusted modified Poisson regression models with robust standard errors.

**Supplemental Table II: Propensity score matching results**

| **Propensity Score Matching model** | | | **INJURY** | | **ASSAULT** | |
| --- | --- | --- | --- | --- | --- | --- |
|  | Dependence | | **(1)**  **PD [95% CI]^a^** | **(2)**  **PD [95% CI]^b^** | **(3)**  **PD [95% CI]^a^** | **(4)**  **PD [95% CI]^b^** |
|  |  | Side job | 0 [0.00, 0.00] | 0 [0.00, 0.00] | 0 [0.00, 0.00] | 0 [0.00, 0.00] |
|  |  | Main job | 0.16*** [0.11,0.20] | 0.13*** [0.08,0.18] | 0.11*** [0.07,0.15] | 0.10*** [0.05,0.15] |
| **Linear Probability Model** | | |  |  |  |  |
|  | Dependence | |  |  |  |  |
|  |  | Side job | 0 [0.00, 0.00] | 0 [0.00, 0.00] | 0 [0.00, 0.00] | 0 [0.00, 0.00] |
|  |  | Main job | 0.15*** [0.11,0.19] | 0.11*** [0.08,0.15] | 0.10*** [0.06,0.13] | 0.07*** [0.03,0.10] |

P value * <0.05 **<0.01 **<0.001

PD= Prevalence difference

Footnotes:

^a^ Matched covariates includes age, gender, race and ethnicity, survey language, and years of employment

^b^ Matched covariates include age, gender, race and ethnicity, survey language, years of employment, mode of transport
